# On Coloring the Lips by Tattooing, after Cheiloplasty

**Published:** 1859-11

**Authors:** 


*On Coloring the Lips by Tattooing, after Cheiloplasty.* By Professor SCHUH. (Wien Medicinische Wochenschrift, 1858, No. 47.)

Two years since Professor Schuh performed cheiloplasty in the Vienna Clinik, upon a girl in whom one-half of the nose, together with the vomer and the whole of both lips, were wanting. The flaps for the lower lip were supplied from the region of the lower jaw and the neck, and that for the nose from the forehead, while the skin of the arm was employed for the upper lip. The connection of the flap with the arm was divided on the tenth day, and all went on well, excepting that the new upper lip, at its lower edge, owing to the cicatricial process, was covered with corion. The red lip-color was wanting to give the mouth an agreeable appearance; and Professor Schuh determined to endeavor to imitate this by tattooing. He first of all tried cochineal as a coloring material, but this produced a too pale red ; and he then had recourse to cinnabar, which gave rise to a surprisingly natural color.

The following is the procedure : the cinnabar is made into a thin paste with water, and the limits within which the pigment is to be applied are traced with a pen and ink, in imitation of the direction of the natural redness of the lips. For forcing the pigment into the organic substance, a bundle of sharp-pointed pins is employed, each pin being wound round with waxed silk from its head to within four lines of its point. Ten or twenty such pins are tied into a bundle with thread, dipped into the coloring substance, and repeatedly forced two or three lines deep into the lip. The margin marked by the ink is first to be colored, and then the other portion, dipping the points into the pigment again as this is wiped off. Only a slight bleeding ensues, and the pain is very little, in consequence of the diminished sensibility of transplanted parts. Any of the pigment remaining on the surface should be left there until next day, and if any part is found to be less red than the others, this can be easily remedied.

How long this redness will remain unchanged must be determined by further experience. In Professor Schuh's case it had become nowise paler at the end of a year and a half; and he believes that the introduction of the process of tattooing into the field of plastic surgery is not to be despised.

